# Development and pilot study of family-centered neonatal palliative care

**DOI:** 10.3389/fmed.2026.1799077

**Published:** 2026-08-26

**Authors:** Na Zhang, Yong Chen, Lihui Zhu, Muhua Chen, Yuee Xiong, Tingwei Luo

**Affiliations:** 1 Early Clinical Research Center, Hunan Cancer Hospital/The Affiliated Cancer Hospital of Xiangya School of Medicine, Central South University, Changsha, China; 2 Department of Nursing, Hunan Children's Hospital, Changsha, China; 3 Department of Neonatology, Hunan Children's Hospital, Changsha, China

**Keywords:** family-centered, neonatal, palliative care, pilot study, qualitative research

## Abstract

**Background:**

The high global neonatal mortality rate, the variety of causes of death, and the poor quality of neonatal survival at the end of life make the implementation of neonatal palliative care (NPC) urgent. Developed countries have developed guidance models and systems for NPC. In developing countries, NPC is still in its infancy, with insufficient practice experience. Therefore, there is an urgent need to develop practice-oriented NPC models.

**Methods:**

Based on the family-centered care (FCC) theory, this study adopted a combination of literature research, qualitative research and the Delphi method to develop a family-centered NPC (FCC-NPC) model. Using a before-and-after control method, 10 cases of end-of-life children in the neonatal department of a children’s specialty hospital in Hunan Province were selected as study subjects to observe the feasibility of model implementation and family satisfaction.

**Results:**

We conducted two rounds of expert correspondence. The recovery rates of the first and second questionnaires were 100%. The average authoritative coefficients were 0.858. The Kendall W values ranged from 0.302–0.503 (*p* < 0 0.01). Six level I indicators, 22 level II indicators, and 66 level III indicators were developed for FCC-NPC. In the pilot study, eight cases were fully completed and two cases were partially completed, for a completion rate of 80.00%. Family satisfaction scores were significantly higher after the pilot study than before the pilot study (*p* < 0.05).

**Conclusion:**

The FCC-NPC developed in this study has good science and practicality, and may provide a reference for NPC clinical practice.

## 1 Introduction

WHO statistics show that the global neonatal mortality rate in 2021 is as high as 17.6 per 1,000 for all live births, with an average neonatal mortality rate of 27 per 1,000 in low-income countries (1), with the main causes of death being prematurity, complications at birth or infectious pneumonia, sepsis, and meningitis (1, 2)^.^ End-of-life neonates with associated symptoms such as pain, respiratory distress and neurological (agitation, sleep disturbances, convulsive seizures) suffer immensely, and their deaths cause great sadness to the entire family (3, 4). When the outcome of neonatal death is unavoidable, the provision of palliative care can alleviate the suffering of terminally ill neonates, improve the quality of their survival in the terminal phase, reduce the psychological burden on the family, and shorten the grieving period (5). Currently, in developed countries, the proportion of newborns receiving palliative care at the end of life accounts for 2.6% of the annual number of newborn deaths, and the rate of hospital-based palliative care services ranges from 1 to 15% (5). However, in developing countries, there is a high demand for neonatal palliative care, but the research on the service rate is still in the exploratory stage. NPC (6) refers to a multidisciplinary collaborative team consisting of medical personnel, social workers, and other related professionals, who provide physiological, psychological, social, and other forms of comprehensive and humanistic care for neonates and their families in the event of unavoidable death. The goal is to minimize the suffering of terminally ill infants, improve their quality of life, reduce the psychological burden on their families, and alleviate negative emotions.

At present, countries such as the United States, the United Kingdom and Australia have been carrying out NPC for more than 20 years, forming a more complete palliative care system, and have successively issued relevant guidelines and feasible programs for NPC. The Medical College of Georgia in the United States developed the Guidelines for Neonatal Comfort/Palliative Care (7), American scholars Caitlin and Carter developed a detailed NPC Program (8), and the National Association of Neonatal Nurses (NANN) updated the Neonatal and Infant Palliative and End-of-Life Care (Position Statement) (9), and the National Institute for Health and Care Excellence (NICE) published Guidelines for the Preparation and Management of Infants, Children and Adolescents in End-of-Life Care (10), etc., which have been widely used as valuable guidance in NPC practice. In addition, developed countries have compiled numerous theoretical and practical research findings across different fields. In Europe and the United States, NPC has been included in health insurance coverage, resulting in a large number of organizations implementing NPC (11). In Germany, the NPC home care team is a multidisciplinary team consisting of neonatologists, nurses, and social workers (12). It is clear from this that in developed countries, NPC is more mature in terms of industry standards, professional practitioners and protection under relevant legislation. However, despite their contributions, these guidelines have limitations when considered for implementation in developing countries. First, they assume dedicated palliative care teams and specialist services, rarely available in low-resource settings. Second, their pharmacological protocols require unrestricted medication access, often unfeasible. Third, they offer limited guidance on culturally adapted family engagement, adopting a Western shared-decision framework. Fourth, they are principle-based rather than operationally specified-they articulate what should be done but lack detail on how to implement within resource constraints. In developing countries, palliative care began later, with the main focus on elderly patients with end-stage diseases and oncology patients, and less emphasis on NPC (12, 13). NPC is still in the early stages of theory and practice and has not yet developed a more mature and systematic management model. Furthermore, the absence of standardized protocols and multidisciplinary team structures across hospitals may contribute to variability in practice, posing challenges to the systematic development of neonatal palliative care in these settings (7). Therefore, it is necessary to develop a scientific and feasible model of NPC.

FCC is a method that involves family members in the entire care process. Its aim is to provide physical, psychological, mental and social comfort for children. FCC is consistent with both the current health-centered care model in the new medical landscape (13) and the core concepts of palliative care. Some countries have well integrated the family-centered care concept into hospital NICUs. Brigham Women’s Hospital, which brings parents and medical staff together for an annual memorial service to mourn deceased newborns. In some hospitals in Canada (14), the medical team makes decisions together with their families about the choice of palliative care programs, the time of death of the child, and the location of the funeral, fully respecting the religious beliefs and values of the child’s family. In China, a family-based pediatric palliative care, Daisy House, has been established to provide palliative care for children and families with terminal tumors. The use of family-centered concepts in these hospitals improves the quality of child survival, reduces the level of depression and grief in the families, and facilitates communication between the families and healthcare professionals. Therefore, based on the theory of family-centered care, this study developed a FCC-NPC model tailored to the context of developing countries. Unlike existing international guidelines (e.g., NANN, NICE), which are designed for well-resourced settings with established palliative care infrastructure, our model addresses common constraints in developing countries, including the absence of dedicated palliative care teams, limited access to specialized medications, and the need for culturally sensitive family engagement strategies. By incorporating locally feasible adaptations-such as task-sharing among existing NICU staff, simplified symptom management protocols, and explicit inclusion of family members as active care partners-we aim to provide a practical and scalable framework for NPC implementation in resource-limited settings. A pilot study was conducted to evaluate its feasibility and acceptability.

## 2 Theoretical foundations

**Figure 1:**
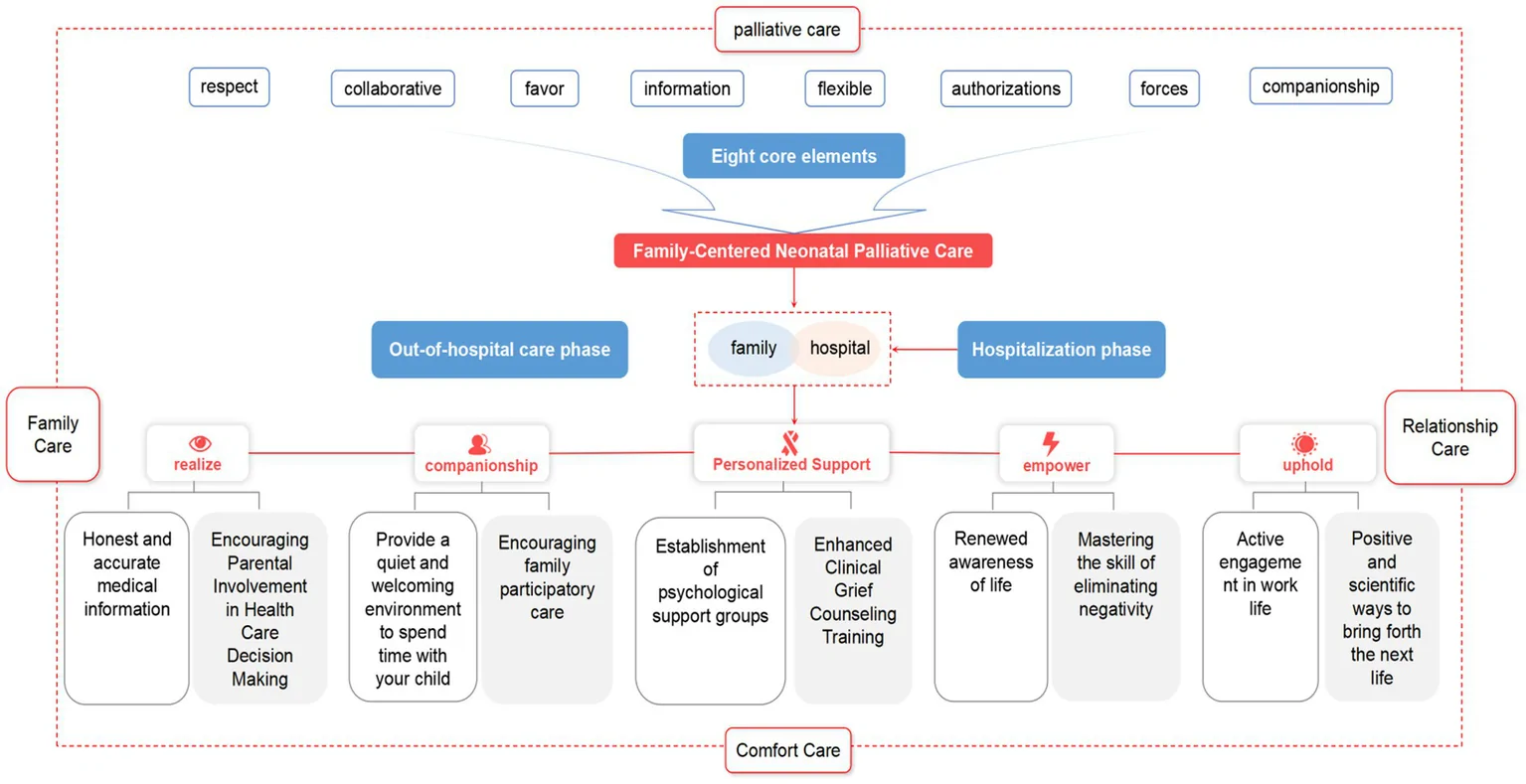
Theoretical framework of family-centered care, illustrating eight cores, two phases, and four categories.

Based on the family-centered care theoretical guidance, it is divided into eight cores, two phases, and four categories (15, 16) (see Figure 1).

## 3 Methods

**Figure 2:**
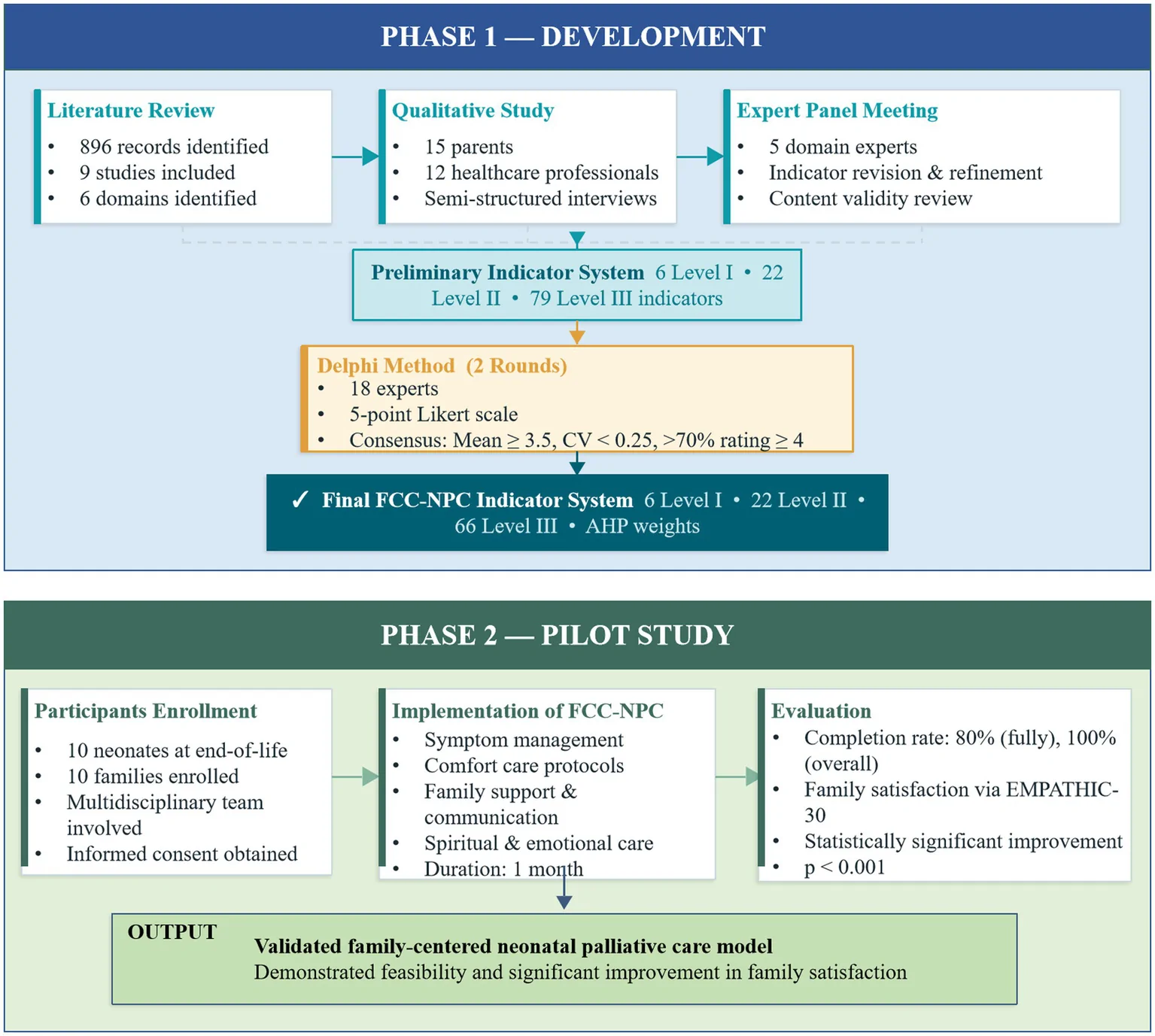
Overall study flow diagram illustrating the two-phase research process.

This study adhered to the Standards for Mixed Methods Article Reporting Standards (MMARS) guidelines (17). A combination of qualitative and quantitative research methods was used to develop FCC-NPC, calculate the weighting coefficients for each level of indicators, and implement a pilot study. The overall research process consisted of two phases: a development phase (literature review, qualitative study, expert panel meeting, and Delphi method) and a pilot phase (implementation and evaluation). An overview of the study design is presented in Figure 2.

### 3.1 The development process of family-centered palliative care for infants

#### 3.1.1 Literature search

Two trained researchers searched the Up To Date, BMJ Best Practice, NICE Cochrane Library, Embase, EBSCO, Medline, Campbell, PubMed, Wiley Online Library, Web of Science databases for eligible studies published from inception to April 2023 using the key words “family-centered/home intervention/family intervention/family based/family therapy”, “infant/newborn/neonate/low birth”, “palliative care/hospice care/end-of-life care.” Indicators associated with FCC-NPC were independently determined by two researchers. Then, two researchers integrated the results, and a third researcher made a decision in the event of different opinions. Inclusion criteria for literature were: (1) studies focusing on neonatal palliative care, end-of-life care, or family-centered care in neonates; (2) study types including clinical guidelines, systematic reviews, meta-analyses, randomized controlled trials, and cohort studies; (3) publications in English or Chinese; (4) full text available. Exclusion criteria were: (1) studies focused solely on adult or pediatric (non-neonatal) palliative care; (2) conference abstracts, editorials, commentaries, or case reports with insufficient methodological detail; (3) duplicate publications; (4) studies not directly relevant to family-centered or neonatal palliative care interventions.

#### 3.1.2 Qualitative study

From May to July 2023, 15 parents of terminally ill newborns and 12 healthcare professionals were selected as respondents for semi-structured interviews. These interviews took the form of telephone interviews and one-on-one face-to-face interviews, and each interview lasted approximately 30–50 min. The outline of the interviews was based on the theory of family-centered care, and the topics of the interviews with parents of newborns were “Is there anything you would like to do for your child?” and “What kind of help would you like to receive from a healthcare professional?” The healthcare provider interview questions were “What would you recommend for developing a family-centered palliative care for newborns?” Literature findings and interviews were integrated to form a preliminary draft, and five experts in the fields of neonatal nursing, neonatal medicine, and palliative care were invited, all of them have the title of associate senior or above. After discussion at the expert group meeting, the names and contents of the indicators, etc., were revised and improved one by one, and the first round of correspondence questionnaires for FCC-NPC was initially formed. Data collection continued until no new themes emerged; thematic saturation was achieved after 23 interviews, with 4 additional interviews confirming existing codes.

#### 3.1.3 Integration of literature and qualitative findings

We integrated literature and qualitative findings to develop the preliminary framework. Two researchers extracted key concepts from nine included studies, organizing 38 evidence items into six domains. Separately, thematic analysis of 27 interviews (15 parents, 12 healthcare professionals) using NVivo 12 yielded four themes: family proximity needs, symptom management priorities, communication needs, and organizational support. Through consensus meetings, we synthesized these sources, identifying convergent areas, complementary insights, and culturally specific gaps. The resulting preliminary indicator system (6 first-level, 22 s-level, and 79 third-level indicators) was reviewed by an expert panel meeting of five senior experts, forming the Delphi questionnaire basis.

#### 3.1.4 Delphi consultation process

From August to September 2023, 18 experts were selected from tertiary A hospitals or nursing schools of national key universities across six provinces (Guangdong, Shanxi, Hunan, Beijing, Shanghai, and Tianjin). Eligible experts held a bachelor’s degree or higher, held titles of associate senior or above, and had at least 5 years of experience in neonatal medicine, neonatal nursing, palliative care, or psychology. Experts comprised: 7 neonatal physicians, 6 neonatal nurses, 3 palliative care specialists, and 2 psychologists. The Delphi process was conducted via email or WeChat, with telephone confirmation of questionnaire content. In the first round, 17 of 18 questionnaires were returned (response rate 94.44%); in the second round, all 18 were returned (response rate 100%). Experts rated the importance of each indicator on a 5-point Likert scale (1 = not important, 5 = very important). Indicators were retained if they met all of the following consensus criteria: mean importance ≥ 3.5, coefficient of variation (CV) < 0.25, and >70% of experts rating the indicator ≥ 4. Indicators not meeting these criteria were considered for deletion, revision, or merging based on expert comments and research team discussion. After the first round, among the 79 third-level indicators initially developed, 12 were deleted, 2 were merged, 2 were modified, and 4 were added, resulting in 66 third-level indicators in the second-round questionnaire (Table 1). Consensus stability was assessed using Kendall’s W, which increased from 0.302 to 0.416 for second-level indicators and from 0.311 to 0.503 for third-level indicators (both *p* < 0.01). Additionally, the proportion of indicators with CV < 0.25 increased from 71.2% in round 1 to 89.4% in round 2. Given the high response rates, achievement of consensus criteria, and stabilization of expert opinions, two rounds were deemed sufficient. After the second round, the analytic hierarchy process (AHP) was applied using mean importance scores to calculate indicator weights. After the second round, the analytic hierarchy process (AHP) was applied using the mean importance scores to construct pairwise comparison matrices. The Satty 1–9 scale was used, and consistency was assessed by calculating the consistency ratio (CR). All CR values were < 0.10, indicating acceptable consistency (18).

**Table 1:** Family-centered neonatal palliative care indicator system with weights.

Level I indicators (weights)	Level II indicators (weights)	Level III indicators	Weights	Coefficient variation
A1 forming the family-centered palliative care team (0.1365)	B1 team members (0.7500)	C1 establishment of multidisciplinary teams of medical, nursing, psychological, pharmacy, anesthesia, physical therapy, social work, hospital management and governmental agencies.	1.0000	0.00
	B2 main duties (0.2500)	C2 Doctors: ① Diagnosis, assessment and treatment of the child’s condition, symptom management, and timely communication with the child’s family to provide information and support ② If a critically ill child is transferred from the general ward, communicate with the physician of the original department and work together to formulate a plan of care that meets the needs of the family and improves the comfort level of the child.	0.0560	7.87
		C3 Nurses: ① Communicate well with the child’s family and continuously assess the care needs of the child’s family ② Work with the physician to develop a care plan that meets the needs of the child’s family and improves the child’s comfort ③ Work with the physician to conduct family conferences ④ Provide family members with emotional support, health education, and education about death ⑤ Provide primary care and environmental setting for the terminally ill child in accordance with the care plan ⑥ Act as a coordinator for members of the multidisciplinary team ⑦ Implement palliative care measures and evaluate and improve them properly	0.2598	0.00
		C4 Pharmacist: ① guide and participate in drug dispensing, preparation ② drug testing and identification ③ participate in the development of clinical drug program	0.1193	10.13
		C5 Counselor: ①Provide psychological counseling and guidance to the family of the child ②Provide grief counseling to family members	0.2360	4.77
		C6 Social workers and volunteers: ① Assess the social resources of the child’s family and actively seek help ② Assist families with financial difficulties to apply for relief funds or social welfare ④ Assist in assessing the wishes of the child’s family ⑤ Assist in grief counseling for the family and funeral guidance	0.0316	5.81
		C7 Hospital management, government agencies: policy-level decision-making	0.2974	4.77
A2 conduct a comprehensive assessment (0.1930)	B3 timing of assessment (0.2297)	C8 When determining that a child has entered the stage of needing palliative care	0.6667	4.77
		C9 Dynamic assessment based on the child’s condition	0.3333	6.61
	B4 assessment method (0.1220)	C10 Conduct and document the assessment using the appropriate assessment tool	1.0000	4.77
	B5 assessment content (0.6483)	C11 Child: ① Assess the child’s family history, past history, and medication history ② Assess whether the condition is life-threatening and affects the child’s lifespan ③ Assess the child’s symptoms such as pain and respiratory distress ④ Assess the child’s mental status, e.g., whether he or she is in a coma, etc. ⑤ Assess the child’s comfort level	0.1667	4.77
		C12 Child’s family: ① Assessment of the child’s family’s desired treatment/care goals ② Attitude of the child’s family toward death ③ Assessment of the child’s family’s psychological condition (anxiety, depression, PTSD, etc.) ④ Assessment of the child’s family’s need for information, communication, and decision-making needs to be assessed, and the formulation to be modified accordingly) ⑤ Family’s economic level ⑥ Social support, such as cultural views, beliefs, and a variety of social relationships (siblings/pets), etc. Spiritual support: values, religious ceremonies, bereavement ceremonies, etc.	0.0333	6.61
A3 setting expected targets (0.0843)	B6 aspects of physical functioning (0.2898)	C13 Relief of symptoms such as pain and dyspnea in the child, increased physical comfort.	1.0000	4.77
	B7 psycho-spiritual (0.5499)	C14 The psychological grief of the child’s family is largely alleviated, and religious beliefs and values are respected.	1.0000	4.77
	B8 social support aspects (0.2402)	C15 Reduced burden and stress of care for families of affected children.	0.5000	6.61
		C16 The needs of the child’s family are largely met, e.g., information support, emotional support, etc.	0.5000	6.61
A4 inclusion of service recipients (0.1013)	B9 inclusion criteria (one of the criteria needs to be met) (0.3333)	C17 ① Specialized children with life-limiting diseases that cannot be cured, such as severe cerebral palsy, neuromuscular degeneration of the brain, and congenital cerebral dysplasia, etc. ② Diseases that are incurable, progressively deteriorating, or have a short survival period (<6 months).	1.0000	6.61
	B10 population served (0.6667)	C18 End-of-life newborns (including premature babies)	0.6667	0.00
		C19 Families of newborns at the end of life	0.3333	6.61
A5 implementation of family-centered palliative care measures (0.2065)	B11 access to information (0.1740)	C20 Mode of access to information: predominantly face-to-face conversations, supplemented by other means (orally, in writing, via SMS text, e-mail or other assistive technologies)	0.3299	6.61

C21 Diversified communication modes: establishing WeChat groups; setting up a communication book for the opinions of children’s families, and providing timely feedback and improvement of the opinions put forward		0.0766	7.87

C22 Doctors and nurses organize regular family meetings to introduce and explain the environment, equipment and personnel, as well as the child’s condition, course, prognosis and treatment options		0.1515	9.76

C23 Information exchange: two-way doctors and nurses/family communication on all matters related to decision-making	0.1700	7.93

C24 Critique: the involvement of doctors, the child’s family and other relevant people in discussing the child’s care plan	0.0578	9.20

C25 Shared decision-making: assisting in the development of a medical care plan that meets the wishes of the child’s family and is dynamically adjusted according to the child’s condition and the wishes of the family.	0.2142	7.93

B12 quality of care (0.1879)	C26 Regularly hold theoretical and skills training and assessment for doctors and nurses to improve the quality of medical and nursing care	0.2500	10.39

	C27 healthcare workers are trained in the basics of palliative care and related concepts	0.5000	6.61

C28 Inclusion of the child’s family in routine or multidisciplinary joint visits	0.2500	12.73

B13 environmental management (0.0837)	C29 Set up a farewell room to create a comfortable, quiet, family-oriented and humanized environment that allows family members to accompany them	0.6586	0.00

	C30 Soft and soothing music for terminally ill children, with sound intensity not exceeding 40 dB	0.1852	12.73

C31 Provision of family waiting areas, etc.	0.1562	7.87

B14 symptom management (0.1599)	*Pain management:* the C32 develops analgesic goals with the child’s family and informs of adverse effects	0.1215	6.61

	C33 Use appropriate pain interventions such as nonpharmacologic and pharmacologic management	0.1228	6.61

	C34 Non-pharmacological interventions: ① The nurse-in-charge guides the family and both are involved in the process, e.g., by using non-nutritive sucking, gentle rocking of the child, skin-to-skin contact, reduction of uncomfortable handling or invasive touching/stimulation, etc. ② Mothers’ voices or heart tones, and soft, soothing music are played for the child in the room.	0.8243	4.77

	C35 Principles of analgesia: ① Follow the wishes of the family, decided by the attending physician and anesthesiologist in consultation, with the lowest dose and maximum effect as the standard of intervention ② Mild pain choose non-steroidal drugs, moderate and severe pain use opioids	0.0657	7.93

	C36 After pain interventions are implemented, analgesic effects need to be observed and recorded	0.1043	6.61

	*Dyspnea:* C37 Perform an assessment: assess the child for respiratory symptoms, including respiratory rate, presence of hypoxia, and respiratory impairment indicators	0.3585	7.93

C38 Measures to be given: ① Follow the wishes of the child’s family and give plans and interventions according to the symptoms of dyspnea	0.2647	11.48

C39 Assist family members with methods such as unblocking the airway	0.6525	11.48

*Neurologic Symptoms:* C40 Evaluate the child for neurologic symptoms, including agitation, convulsive seizures, and disorders of consciousness (e.g., coma), and healthcare professionals should closely monitor for signs of agitation, coma, and convulsions.	0.0753	11.48

C41 Follow the wishes of the child’s family and use medications such as anticonvulsants and opioids to aid in sedation	0.0664	12.17

C42 Instruct the child’s family to participate by placing the child in a secure position, performing back rubs, or giving a pacifier	0.2640	11.48

B15 comfort care (0.1404)	C43 Oral care: ① assess the condition of the child’s oral mucosa and designate individualized oral care interventions to promote oral comfort ② keep the mouth moist and clean, and clean the mouth at least 4 times a day with infant mouthwash, 0.9% sodium chloride solution, or chlorhexidine cotton balls	0.9381	11.48

	C44 Skin care: keep the skin clean, clean the child’s body as needed, give the child a full-body bath once a day, moisturize the whole body with moisturizing cream, avoid wearing tight-fitting clothes or clothes made of materials that do not absorb perspiration, and change the child’s clothes and bedclothes in a timely manner.	0.8953	7.93

C45 Sleep care: day and night nursing operations try to concentrate, gentle movements, timely handling of monitor alarms, to create a good sleep environment for the child	0.2761	6.61

C46 excretion care: ① assess the child’s excretion, for children with diarrhea, to clean up the excreta in a timely manner, to increase the sense of comfort ② prevention of constipation, the child’s bowel movements, can be prevented by the use of laxatives to relieve constipation.	0.3905	6.61

B16 psychological interventions (0.0751)	C47 Need for dynamic assessment of psycho-emotional responses, caregiving burden and stress, etc.	0.5000	7.93

C48 Counselors take the initiative to seek the opinions of the child’s family, convey empathy, listen to the family’s fears, hopes, pain, and burden of care, provide timely one-on-one psychological counseling for families with negative emotions, and instruct families to write diaries to relieve anxiety	0.5000	7.93

B17 social support (0.0425)	C49 Assess the support of the child’s family (e.g., financial support, etc.)	0.2500	8.95

	C50 medical and nursing staff and social work department staff communicate with families of terminally ill children to help cope with negative emotions and provide personalized psychological support, such as keeping commemorative items such as rim full boxes, family photos, baby handprint cards, footprint cards, etc., and engaging in physical contact such as hugging and caressing with the affected children, so that the parents will have the opportunity to express their sense of loss and pain.	0.5000	6.61

C51 Medical social workers consolidate policies related to health insurance and information on financial assistance from the government and the private sector, provide information support and assist families with financial difficulties in applying for social welfare, etc.	0.2500	8.95

B18 death education (0.1951)	C52 Assess the child’s family’s attitudes toward death and provide targeted answers and counseling	0.6667	6.61

C53 Meeting Spiritual Needs Suggests that families express love, do the Four Ways of Life, i.e., Apologize, Thank, Love, and Farewell, and help families rebuild hope.	0.3333	12.73

B19 grief counseling (0.0414)	C54 Support during the acute grieving period: companionship, care of the body, burial with meaningful objects	0.0656	9.20

	C55 Assess the child’s family’s expectations of the timing, frequency, and format of grief follow-up visits, respecting the child’s family’s choices	0.2625	7.93

	C56 Instruct the child’s family to watch and read movies and books related to grief therapy to encourage emotional release	0.2864	9.76

C57 Instruct the family to organize photographs, texts and mementos about the deceased, and also to honor the child with texts, photographs, videos, etc.	0.1473	10.77

C58 Families of children with severe depression, anxiety, and other maladies, help families refer to psychiatric mental health centers	0.2037	6.61

C59 used the Family Dignity Intervention, a Family Dignity Therapy interview outline that used three themes of looking back, focusing on oneself, and looking forward to the future to layer in order to promote family closeness	0.0345	7.87
A6 formation of an evaluation system (0.2784)	B20 evaluation objects (0.3077)	C60 Families of children at the end of life	0.3012	7.93
		C61 Children at the end of life	0.6264	6.61
		C62 Medical team	0.0724	7.87
	B21 evaluation timing (0.0769)	C63 Pre-, mid- and postimplementation process	1.0000	6.61
	B22 evaluation content (0.6154)	C64 Families of children at the end of life: anxiety, level of depression, satisfaction, burden of care, basic needs	0.2500	7.93
		C65 Children at the end of life: pain, respiratory distress, neurological symptoms, etc.	0.5000	6.61
		C66 The medical team: empathic ability	0.2500	7.93

### 3.2 Pilot study process of family-centered palliative care for infants

To further validate the feasibility of FCC-NPC, this study used a before-and-after control method to sample 10 neonates and their families, who met the inclusion and exclusion criteria, from the neonatal department of a children’s specialty hospital in Hunan Province between October 2023 and March 2024.

#### 3.2.1 Pilot study participants

Inclusion criteria for newborns: ① children with a clear diagnosis of incurable life-limiting diseases/children with incurable diseases, progressive deterioration, or short survival period (<6 months); ② family members willing to accept palliative care; ③ children at the end of life aged 0–28 days. Inclusion criteria for neonatal family members: ① family members of children aged 0–28 days at the end of life; ② no history of mental illness, no psychological and cognitive dysfunction, and no serious organic disease; ③ informed consent and in accordance with ethical principles. Exclusion criteria: ① dead newborns with hospitalization time <3 h and their families.

#### 3.2.2 Pilot study implementation

First, a pilot study implementation team was established. The team consisted of eight members: one neonatologist, one neonatal nurse, one counselor, one pharmacist, one anesthesiologist, one physical therapist, one social worker, and one hospital administrator. The neonatologist was responsible for medical decision-making and symptom management; the neonatal nurse coordinated daily care, comfort measures, and family support; the counselor provided psychological support and grief counseling to families; the pharmacist advised on medication management, including analgesia and sedation; the anesthesiologist assisted with pain and symptom control; the physical therapist provided comfort-oriented positioning, gentle touch, and developmental support; the social worker coordinated community resources, facilitated advance care planning, and arranged bereavement follow-up; and the hospital administrator supported resource allocation, operational coordination, and policy adherence.

Team members were trained by the principal investigator over 1 week (1 hour per day) on the study objectives, neonatal palliative care principles, implementation of the FCC-NPC protocol, and the use of evaluation instruments. Training effectiveness was verified through operational and theoretical assessments.

The implementation protocol allowed families to accompany neonates throughout the end-of-life period. Based on the finalized FCC-NPC indicator system (Table 1), multidisciplinary team members were responsible for environmental preparation, symptom management, comfort care, and family emotional support. The pilot study lasted 1 month for each enrolled neonate.

#### 3.2.3 Evaluation indicators

General information was self-designed and included basic characteristics of end-of-life neonates (e.g., gender, gestational age, age in days at admission, weight at admission, and birth information) as well as basic characteristics of their family members (e.g., gender, age, literacy level, religious beliefs, and place of residence).

Feasibility evaluation of FCC-NPC: Feasibility refers to the completion of FCC-NPC by the families of the children and the healthcare workers. In this study, the completion of FCC-NPC was categorized into three levels: *Fully completed:* all planned indicators of the FCC-NPC protocol were implemented; *Partially completed:* more than one-third but not all of the planned indicators were implemented; *Incomplete:* one-third or fewer of the planned indicators were implemented. To evaluate feasibility, we calculated the overall implementation rate, defined as the proportion of cases in which at least partial completion was achieved (i.e., fully or partially completed cases) among the total enrolled cases. In addition, we reported the full completion rate separately to reflect the proportion of cases in which the entire protocol was successfully delivered.

Satisfaction of families after participating in the care process: the study evaluated the satisfaction of families after they participated in FCC-NPC by using the Evaluation of Parental Satisfaction in NICU Scale (empowerment of parents in the Intensive Care, EMPATHIC-30) (19). The scale was developed by Jos. Latour’s team at the University of Plymouth, UK, and Chineseized by the neonatal team at Hunan Children’s Hospital. All entries in the scale are scored on a 6-point scale, with a total score range of 30–180 points, with higher scores indicating greater satisfaction. The scale has good reliability with Cronbach’s alpha of 0.95.

#### 3.2.4 Data collection

During the pilot study, all the terminally ill newborns were provided with the FCC-NPC service in their own independent rooms in the NICU, with no other infants present. The research content and purpose of the study were explained to the study participants, and after the study participants signed the informed consent form, they were surveyed using the General Information Questionnaire, Family-Centered Neonatal Palliative Care Completion Rate, and the EMPATHIC-30. Satisfaction of the study subjects was assessed again at the end of the 1-month pilot study. Before completion, it was explained to the study subjects that the survey was anonymous and anonymous, and they completed the questionnaires independently, and they were filled out and returned on-site, with a 100% return rate and each questionnaire was valid.

### 3.3 Statistical analysis

IBM SPSS 25.0 was used to calculate the coefficients of motivation, authority and coordination of experts to test the reliability of experts. The 95% CI for the median difference was calculated using the Hodges-Lehmann estimator. All statistical tests were two-sided. AHP 10.3 statistical software was used to calculate the weights of the indicators. Categorical data were described using frequency and composition ratios, whereas continuous data were expressed as the median and interquartile range. Comparisons before and after the pilot study were made using paired nonparametric tests. Continuous data were assessed for normality using the Shapiro–Wilk test. As the pilot study sample size was small (*n* = 10) and the data were not normally distributed, nonparametric tests were employed. Comparisons before and after the pilot study were made using the Wilcoxon signed-rank test. Categorical data were described using frequencies and percentages. A two-tailed *p* < 0.05 was considered statistically significant.

## 4 Results

### 4.1 The results of the literature study

**Figure 3:**
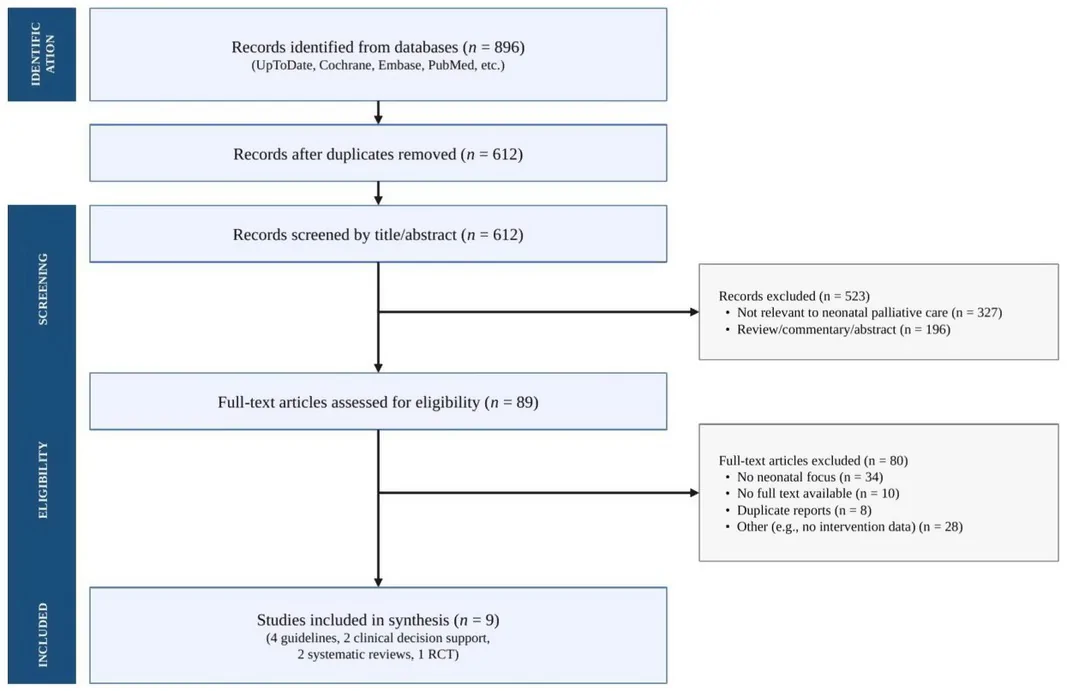
Flowchart of literature screening.

A total of 896 documents were searched by two independent researchers. After exclusion of literature with incompatible research subjects, reviews, and incompatible research topics, nine pieces of literature remained. The quality evaluation of the nine literatures showed that all of them had a low risk of bias and were finally included. Including 4 guidelines, 2 clinical decision-making articles, 2 systematic evaluations, and 1 RCT, as shown in Figure 2. The evidence topics include forming a palliative care team, conducting a comprehensive assessment, setting desired goals, including service recipients, implementing palliative care measures, and forming an evaluation system. The content of the evidence includes the assessment of the family history, past medical history, medication history of the child, pain assessment and management of newborns, medication guidelines, and the involvement of family members in accompanying terminally ill children, etc. Literature research has revealed that the needs of parents of terminally ill newborns and the professional assessment and management content provided by medical staff in the neonatal department are incomplete. Further qualitative research is needed to summarize these aspects (Figure 3).

### 4.2 Qualitative research results

A total of 15 parents of newborns and 12 healthcare professionals were interviewed for the qualitative study. The interviews with parents of newborns yielded their need to be close to their end-of-life newborns, such as hugging the child, being with the child, and breastfeeding the child. Interviews with healthcare professionals yielded that there are needs for pain relief, respiratory management, and comfort care for neonates at the end of life, and that there is a need for a palliative care multidisciplinary team. The results of the preliminary literature study and qualitative study were combined with the results of the discussion at the expert panel meeting. Based on the supplementary results obtained from qualitative research, finally, a questionnaire with 6 level 1 indicators, 22 level 2 indicators and 79 level 3 indicators was developed for the next Delphi study.

### 4.3 Result of expert inquiry

#### 4.3.1 The enthusiasm of experts

The enthusiasm of the experts can be expressed by the recovery rate of the questionnaires. A total of 18 questionnaires were sent out in the first round of questioning and 17 were recovered, a recovery rate of 94.44%. In the second round, 18 questionnaires were sent out and 18 were recovered, with a recovery rate of 100%.

#### 4.3.2 The authority of experts

The degree of expert authority is the arithmetic mean of the expert’s basis of judgment and familiarity, denoted by Cr. In the study, the Cr of consultation experts was 0.858.

#### 4.3.3 Degree of coordination of expert opinions

The degree of harmonization of expert opinions is expressed through Kendall’s W. A larger W represents a better degree of harmonization of expert opinions (20). The Kendall’s W coefficients for the two rounds of expert consultation on level 2 indicators were 0.302 and 0.416, respectively, and those for level 3 indicators were 0.311 and 0.503, respectively. Chi-square test showed that there was a statistical significance (*p* < 0.01).

#### 4.3.4 Revision, weight and consistency test of evaluation indicators at all levels

After the expert survey and statistical analysis of the data, the experts unanimously determined that of the second-level indicators, three were modified. For example, experts suggested changing “condition guarantee” to “care quality,” the suggestion was adopted after discussion by the research team; of the third-level indicators, two were merged, two were modified, 12 were deleted and four were added. For example, there are 4 experts who question whether “healthcare workers” should be classified as a service population? Regarding the significance of being an object? It is not recommended to include these experts, so they have been deleted. The consistency test was performed on the judgment matrix, and the λmax value of the first-level indicator was 6.1242, the CR value was 0.0200, and the consistency test CR < 0.1; the λmax values of the second-level indicators were 2.0000, 3.0037, 3.0183, 2.0000, 9.4794, and 3.0000, respectively; the CR values were 0.0000, 0.0032, 0.0158, 0.0000, 0.0413, and 0.0000, and the consistency test CR are <0.1, which indicates that the expert judgment matrix has high consistency and the results of the study are more reliable.

#### 4.3.5 Determining the family-centered palliative care for infants

Family-centered palliative care for newborns was finalized, including 6 first-level indicators, 22 s-level indicators, and 66 third-level indicators. Content covers establishing an FCC palliative care team, including team composition and the responsibilities of different members; conducting comprehensive assessments, including assessment timing, methods, and content; setting expected goals, including physical function goals, psychological and spiritual goals, and social support goals; incorporating FCC service recipients, including eligibility criteria and an introduction to the target population; Implementing FCC palliative care measures, including information gathering, quality of care, environmental considerations, symptom management (pain management, dyspnea, and neurological symptoms), comfort care, psychological interventions, social support, death education, and grief counseling; Finally, establishing an evaluation system, encompassing evaluation subjects, timing, and content. At the same time, the weights and coefficients of variation for each level of indicators were determined based on the experts’ assignment of importance to each level. The complete indicator system is presented in Table 1.

### 4.4 Pilot study results

#### 4.4.1 General information results of the study subjects

Ten children and their parents in the neonatal department of a children’s hospital in Hunan Province were selected as study subjects (Table 2). Among them, 6 (60.00%) were baby boy and 4 (40.00%) were girl; the gestational age ranged from 33.5 to 40.3 weeks, with an average gestational age of (37.03 ± 2.65) weeks; the birth weight ranged from 1,450 to 4,000 g, with an average of (2,791.00 ± 823.17) g. Detailed information on the general data of the research subjects is shown in Tables 3, 4.

**Table 2:** The judgment matrix and weight of level I indicators.

Index	A1 forming the family-centered palliative care team	A2 conduct a comprehensive assessment	A3 setting expected targets	A4 inclusion of service recipients	A5 implementation family-centered palliative care measures	A6 forming an evaluation system	Weight
A1 forming the family-centered palliative care team	1	1	2	1	1/2	1/2	0.137
A2 conduct a comprehensive assessment	1	1	2	2	1	1	0.193
A3 setting expected targets	1/2	1/2	1	1	1/3	1/3	0.084
A4 inclusion of service recipients	1	1/2	1	1	1/2	1/3	0.101
A5 implementation of family-centered palliative care measures	2	1	3	2	1	1/2	0.207
A6 forming an evaluation system	2	1	3	3	2	1	0.278

**Table 3:** Basic characteristics of newborns at the end of life (*n*, %).

Projects	Sub-categories	Number of cases (*n*)	Composition ratio (%)
Genders	Baby boy	6	60.00
	Girl	4	40.00
Gestational age (weeks)	37.03 (33.50, 40.30)		
Age at admission (d)	6 (0.5, 21)		
Weight at admission (g)	2791.00 (1450.00, 4000.00)		
Circumstances of birth	Abnormalities of the umbilical cord (umbilical cord wrapped around the neck, prolapsed, knotted)	2	20.00
	Abnormalities of the placenta (placenta previa, placenta previa, sail)	6	60.00
	Amniotic fluid abnormalities (too much, too little, contamination)	1	10.00
	Birth asphyxia	1	10.00
Diseases that cause the child to be critically ill	Septicaemia	3	30.00
	Birth defect	2	20.00
	Severe pneumonia	2	20.00
	Necrotizing small bowel colitis	2	20.00
	Neonatal respiratory distress syndrome (NRDS)	1	10.00

**Table 4:** Basic characteristics of families of newborns at the end of life (*n*, %).

Projects	Sub-categories	Number of cases (*n*)	Composition ratio (%)
Genders	Male	3	30.00
	Female	7	70.00
Age (years)	<25	2	20.00
	25–45	3	30.00
	>45	5	50.00
Educational attainment	Junior high school and below	2	20.00
	Senior high school	4	40.00
	University and higher	4	40.00
Religious belief	None	8	80.00
	Buddhist	2	20.00
Per capita monthly income (yuan)	<2,000	2	20.00
	2,000–4,999	2	20.00
	>5,000	6	60.00
Residence	Cities	4	40.00
	Countryside	6	60.00
Medical insurance reimbursement	City and town medical insurance	4	40.00
	Rural health insurance	5	50.00
	Self-funded	1	10.00

#### 4.4.2 Evaluation of the effect of family-centered neonatal palliative care pilot study

Evaluation of the feasibility of FCC-NPC: the feasibility of FCC-NPC was evaluated after a 1-month implementation of FCC-NPC for 10 terminally ill neonates. The results showed that among the 10 enrolled cases, 8 were fully completed and 2 were partially completed, yielding an overall implementation rate of 100% and a full completion rate of 80.00%.

Evaluation of satisfaction with FCC-NPC: the satisfaction score was significantly higher after the pilot study (148.00 ± 8.12) than before (122.10 ± 13.52), with a median difference of 25.90 (95% CI: 15.20–36.60), *Z* = −3.516, *p* < 0.001. Among them, the content of the model that the family members were most satisfied with was the retention of baby handprint cards and other commemorative items as part of social support, as well as physical contact such as hugs and gentle caresses with the child, with a satisfaction rate of 100%. The least satisfactory content of the model was the occurrence of neurological symptoms in symptom management, and following the wishes of the child’s family and using medications such as anticonvulsants and opioids to aid in sedation, with a satisfaction rate of 35%.

## 5 Discussion

As a pilot feasibility study, this research was designed to test implementation processes and acceptability, not to establish definitive intervention effects. In developing the FCC-NPC, this study was guided by the theory of family-centered care and incorporated evidence from relevant guidelines and research through a systematic literature search. Meanwhile, combining the results of semi-structured interviews with 15 family members of terminally ill newborns and 12 healthcare professionals in the preliminary stage, the key elements and priorities of FCC-NPC were summarized to provide a scientific basis for its development. In addition, this study revised and improved the developed FCC-NPC through 2 rounds of expert consultation, and 18 consulting experts were selected from 6 provinces (cities), involving neonatal medical or nursing care, psychology, and other work fields closely related to palliative care, so that reliable and professional revision opinions could be obtained from different perspectives. In the 2 rounds of consultation, the effective questionnaire return rate was 94.44 and 100%, indicating that the experts were highly motivated; the Cr of the experts was 0.858, suggesting a high degree of authority and good representativeness; and the Kendall’s W of the entries at all levels was 0.302–0.416 and 0.311–0.503, respectively (both *p* < 0.01), indicating that the degree of harmonization of the experts’ opinions was good. After two rounds of expert correspondence, the weights of the first-, second- and third-level entries were calculated using hierarchical analysis based on the mean scores of the entries after the second round of expert consultation. Compared with the Catlin & Carter protocol and NANN guidelines, which are principle-based and assume specialist teams, our model provides operationalized indicators adapted to resource-limited settings through task-sharing and simplified protocols. The weight coefficients of “A6 Forming an evaluation system, A5 Implementing family-centered palliative care measures, and A2 Conducting a comprehensive assessment” in the first-level entries are 0.2784, 0.2065, and 0.1930, respectively, which are the three most important entries. Among the secondary indicators, the weight coefficients of B22 evaluation content and B20 evaluation object were 0.6154 and 0.3077, respectively, which showed that the evaluation object and content were relatively important; the weight coefficients of B14 symptom management, B15 comfort care, and B13 environmental management were 0.1599, 0.1404, and 0.0837, respectively, which indicated that symptom management, comfort care, and environmental management are very important in the implementation of NPC. Finally, the developed FCC-NPC was piloted in a pilot study, and the multidisciplinary palliative care team strictly followed each indicator of NPC implementation and evaluated its feasibility and satisfaction. In conclusion, the development process appears methodologically sound, as supported by the Delphi consensus indicators.

The development of NPC is influenced by a variety of factors, including healthcare organizations, healthcare professionals, families, and social culture (21). Following the principles of comprehensiveness, hierarchy, and feasibility, this study developed a FCC-NPC in conjunction with a hospital clinical scenario. The framework comprises six components: establishing a family-centered palliative care team, conducting a comprehensive assessment, defining care goals, engaging family members, implementing family-centered care measures, and establishing an evaluation system. The American Academy of Pediatrics and related experts have recommended (22) that the palliative care team be composed of physicians, nurses, social workers, and other professionals in order to provide complete and continuous services. Currently, most healthcare organizations in developing countries have not yet formed palliative care teams, and there is no standard for their composition. In this study, clinicians, clinical nurses, social workers, and family members are considered as the main members, and it is also recommended that healthcare organizations add support staff such as psychologists and pharmacists as needed according to the illnesses of end-of-life newborns and their families, with the aim of providing a more comprehensive and professional service to newborns and their families. Regarding the target population to be included in FCC-NPC, several guidelines have stated (23, 24) that children with special needs who have incurable life-limiting illnesses, such as severe cerebral palsy, or children with incurable, progressively worsening diseases or short survival periods (<6 months) are among the criteria for admission to NPC. Symptom management, comfort care, and environmental management are all core components in the implementation of family-centered palliative care measures (25), and are major factors affecting the quality of life of neonates at the end of life. Pain management in symptom management is an urgent challenge in NPC. Therefore, it is recommended that healthcare professionals select appropriate pain assessment tools based on the gestational age of the neonate, specific clinical scenarios, and sequentially select analgesic measures of different intensities based on the degree of pain and in compliance with the family’s wishes to alleviate neonatal suffering. In addition, malnutrition in end-of-life neonates is easily overlooked, and healthcare professionals are recommended to carry out individualized nutritional management based on the neonate’s sucking and swallowing, digestive function, feeding tolerance, and expected survival time, as well as combining with the wishes of his/her family (26). Enhanced oral care is a critical component of comfort care. In end-of-life neonates, symptoms of thirst are more closely related to oral mucosal dryness than fluid intake (3). Through 2 rounds of correspondence, the coefficient of variation for all entries was <25. After implementing a 1-month FCC-NPC in 10 end-of-life neonates through a pilot study, 8 completed the NPC in its entirety, and 2 partially, for a completion rate of 80.00%, which is good for feasibility. In conclusion, The pilot findings suggest that FCC-NPC demonstrates preliminary feasibility and acceptability. While completion rate served as a primary indicator of feasibility in this pilot study, we acknowledge that feasibility is a multidimensional construct citation. Future large-scale studies should incorporate mixed-methods evaluations, including qualitative interviews and structured acceptability scales, to capture these dimensions more comprehensively.

Most critically ill newborns are sent to the hospital at birth, and some die directly in the hospital, turning the joy of welcoming a newborn into the grief of losing a child, which is often difficult for the family to accept. This serious negative event has a huge impact on the family (27), and the direct immediate effects mainly include various negative emotions such as sadness, depression, anxiety, self-blame, shame, etc. Some parents attribute the death of their child to their own faults and harbor profound guilt and self-reproach (28). Long-term consequences may include adverse health behaviors such as alcoholism and drug dependence, and even suicidal tendencies. When the outcome of neonatal death is unavoidable, the focus of their care should be shifted from aggressive therapeutic interventions to soothing palliative care, which can be highly effective in relieving the child’s pain and reducing the family’s grief (5). In this study, after the implementation of FCC-NPC, the satisfaction of the families of the children increased significantly compared with the pre-implementation period, which is consistent with the findings of De Zen and Hamel (4, 29). The reason for this may be that FCC-NPC focuses on the cooperation between palliative care team members and the families of the newborns, fully respects the decision-making power of the families, takes into account the wishes of the families when implementing palliative care measures, and allows the families to be in close contact with their children, and therefore, the families of the affected children expressed great satisfaction and gratitude. Therefore, the FCC-NPC framework developed in this study is clearer, more specific and practical, and offers guidance for the clinical practice of NPC in developing countries.

### 5.1 Strengths and limitations

There were several strengths to our study. First, the area studied was palliative care, which has been the focus of attention in recent years, and in conjunction with the bias of palliative care emphasis on populations, we shifted our attention to the neonatal population, which rarely receives attention from palliative care, and found that there is very little standardized guidance on clinical practice for NPC in developing countries. Second, FCC-NPC was developed through a combination of subjective and objective approaches, and its science, feasibility, and utility were validated through a pilot study. Several limitations should be acknowledged. The small sample size (*n* = 10) and absence of a control group preclude causal inference and limit statistical power. The Hawthorne effect may have inflated satisfaction scores. Outcome assessment was restricted to feasibility and satisfaction; future research should include clinical outcomes (e.g., pain, quality of dying), parental psychological measures, and cost evaluations.

To the best of our knowledge, this study represents one of the first efforts to systematically develop and pilot a FCC-NPC model in mainland China, with explicit consideration of the resource and cultural contexts of developing countries. The model demonstrates good scientific rigor and practical feasibility. Hospital administrators and clinicians in similar settings may adapt the proposed indicators to their local circumstances to advance the development of neonatal palliative care in regions where such services remain limited.

## Data Availability

The original contributions presented in the study are included in the article/supplementary material, further inquiries can be directed to the corresponding author/s.

## References

[1] World Health Organization. World Health Statistics. (2021). Monitoring health for the SDGs. Available online at: https://www.who.int/data/gho/publications/world-health-statistic (Accessed January 2, 2022).

[2] Hug L You D Blencowe H Mishra A Wang Z Fix MJ . Global, regional, and national estimates and trends in stillbirths from 2000 to 2019: a systematic assessment. Lancet. (2021) 398:772–85. doi: 10.1016/S0140-6736(21)01112-0 34454675 PMC8417352

[3] Garten L Bührer C. Pain and distress management in palliative neonatal care. Semin Fetal Neonatal Med. (2019) 24:101008. doi: 10.1016/j.siny.2019.04.008, 31056417

[4] Hamel MN Beltran SJ. Factors that contribute to bereaved parents' perceptions of neonatal palliative care: a systematic literature review. Am J Hosp Palliat Care. (2023) 40:658–68. doi: 10.1177/10499091221113277, 35793131

[5] Dombrecht L Piette V Deliens L Cools F Chambaere K Goossens L . Barriers to and facilitators of end-of-life decision making by neonatologists and neonatal nurses in neonates: a qualitative study. J Pain Symptom Manag. (2020) 59:599–608.e2. doi: 10.1016/j.jpainsymman.2019.10.007, 31639496

[6] Roeland EJ Lindley LC Gilbertson-White S Saeidzadeh S Currie ER Friedman S . End-of-life care among adolescent and young adult patients with cancer living in poverty. Cancer. (2020) 126:886–93. doi: 10.1002/cncr.32609, 31724747 PMC6992488

[7] Carter BS Bhatia J. Comfort/palliative care guidelines for neonatal practice: development and implementation in an academic medical center. J Perinatol. (2001) 21:279–83. doi: 10.1038/sj.jp.7210582, 11536019

[8] Catlin A Carter B. Creation of a neonatal end-of-life palliative care protocol. J Perinatol. (2002) 22:184–95. doi: 10.1038/sj.jp.7210687, 11948380

[9] NANN. Palliative and End-of-Life Care for Newborns and Infants: Position Statement. Schaumburg: National Association of Neonatal Nurses (2015).

[10] NICE. End of Life Care for Infants, Children and Young People with Life-Limiting Conditions: Planning and Management. London: National Institute for Health and Care Excellence (2016). 28045476

[11] Section on Hospice and Palliative Medicine and Committee on Hospital Care. Pediatric palliative care and hospice care commitments, guidelines, and recommendations. Pediatrics. (2013) 132:966–72. doi: 10.1542/peds.2013-2731, 28448256

[12] Adler K Schlieper D Kindgen-Milles D Meier S Schwartz J van Caster P . Integration of palliative care into intensive care: Systematic review. Anaesthesist. (2017) 66:660–6. doi: 10.1007/s00101-017-0326-0, 28589374

[13] Uniacke S Browne TK Shields L. How should we understand family-centred care. J Child Health Care. (2018) 22:460–9. doi: 10.1177/1367493517753083, 29347833

[14] Ward V Freeman S Banner D. Hospice care provider perspectives of medical assistance in dying in a Canadian hospice that does not provide medical assistance in dying. Can J Nurs Res. (2022) 54:3–14. doi: 10.1177/0844562120985995, 33435718

[15] Silverman WA. A hospice setting for humane neonatal death. Pediatrics. (1982) 69:239–40. doi: 10.1542/peds.69.2.239, 7058104

[16] McDermott CL Engelberg RA Woo C. Novel data linkages to characterize palliative and end-of-life care: challenges and considerations. J Pain Symptom Manag. (2019) 58:851–6. doi: 10.1016/j.jpainsymman.2019.07.017, 31349037 PMC6823151

[17] Levitt HM Bamberg M Creswell JW Frost DM Josselson R Suárez-Orozco C. Journal article reporting standards for qualitative primary, qualitative meta-analytic, and mixed methods research in psychology: the APA publications and communications board task force report. Am Psychol. (2018) 73:26–46. doi: 10.1037/amp0000151, 29345485

[18] Park S Kim HK Lee M. An analytic hierarchy process analysis for reinforcing doctor-patient communication. BMC Prim Care. (2023) 24:24. doi: 10.1186/s12875-023-01972-3, 36670353 PMC9860231

[19] Zhuang Y Zhang R Gao XR Zhu LH Latour JM. Validation of the Chinese empowerment of parents in the intensive care (EMPATHIC-30) questionnaire among parents in neonatal intensive care units: a prospective cross-sectional study. Front Pediatr. (2022) 10:851291. doi: 10.3389/fped.2022.851291, 35433534 PMC9005953

[20] Nasa P Jain R Juneja D. Delphi methodology in healthcare research: how to decide its appropriateness. World J Methodol. (2021) 11:116–29. doi: 10.5662/wjm.v11.i4.116, 34322364 PMC8299905

[21] Li FX Liu ZZ Hu CW Zhu XM Wang XQ Sun J . Construction of a death education program for nursing staff based on constructivism learning theory. Chin J Nurs. (2021) 56:1685–90. doi: 10.3761/j.issn.0254-1769.2021.11.014

[22] American Academy of Pediatrics. committee on Bioethics and committee on Hospital Care. Palliative care for children. Pedatics. (2000) 106:351–7. 10920167

[23] National Institute for Health and Care Excellence (NICE). End of Life Care for Infants, Children and Young People with Life-Limiting Conditions: Planning and Management. [EB/OL]. London: National Institute for Health and Care Excellence (NICE) (2016). http://www.nice.org.uk/guidance/ng61 28045476

[24] Department of Health & Children. Palliative Care for Children with Lifelimiting Conditions in Ireland. [EB/OL]. Dublin: Department of Health & Children (2010). https://www.gov.ie/en/publication/ed697f-palliative-care-for-children-with-life-limiting-conditions-in-irelan/

[25] Cheng R Yang Y. Expert consensus on neonatal pain assessment and analgesia management (2020 edition). Chin J Contemp Pediatr. (2020) 22:923–30. doi: 10.7499/j.issn.1008-8830.2006181 PMC7499443 32933620

[26] Zhao LH Wang XL Zhu YJ Pu QT Hu YL Shi ZY . Application of behavioral cue-based oral feeding in the change of preterm infants feeding concept in NLCU. Chin J Nurs. (2022) 57:1013–8. doi: 10.3761/j.issn.0254-1769.2022.08.019

[27] Liu Y Zhao MH. Caring for families experiencing perinatal loss: a literature review. Chin J Nurs. (2019) 54:125–30. doi: 10.1016/j.siny.2019.101008

[28] Garstang Y Griiths F Sidebotham P. What do bereaved parents want from professionals after the sudden death of their child: a systematic review of the literature. BMC Pediatr. (2014) 14:269. doi: 10.3761/j.issn.0254-1769.2019.01.024 25319926 PMC4287432

[29] De Zen L Del Rizzo I Ronfani L Barbieri F Rabusin M Dall'Amico R . Safety and family satisfaction of a home-delivered chemotherapy program for children with cancer. Ital J Pediatr. (2021) 47:43. doi: 10.1186/1471-2431-14-269 33637114 PMC7908006

